# Retalho cutâneo medial da coxa irrigado por vasos perfurantes do pedículo secundário do músculo grácil: Estudo anatômico e relato de caso clínico

**DOI:** 10.1055/s-0045-1809523

**Published:** 2025-07-15

**Authors:** Gabriel Vique Valeriano, Antonio Carlos da Costa, Diego Figueira Falcochio, Yussef Ali Abdouni

**Affiliations:** 1 Grupo de Cirurgia da Mão e Microcirurgia, Departamento de Ortopedia e Traumatologia, Irmandade da Santa Casa de Misericórdia de São Paulo (DOT/ISCMSP), São Paulo, SP, Brasil

**Keywords:** coxa, músculo grácil, retalhos cirúrgicos, gracilis muscle, surgical flaps, thigh

## Abstract

**Objetivo:**

Estudar a anatomia dos vasos perfurantes que irrigam a pele da face medial da coxa, provenientes do pedículo vascular secundário do músculo grácil.

**Métodos:**

Foram dissecadas 33 coxas de cadáveres de ambos os sexos, e registradas e analisadas as características dos vasos sanguíneos que irrigam o músculo grácil no terço médio.

**Resultados:**

Em todas as coxas (100%) foi evidenciada a presença de um pedículo vascular secundário no septo intermuscular entre os músculos grácil e vasto medial/sartório, com ramos que irrigam a pele suprajacente na face medial. O pedículo apresentou um diâmetro arterial que variou de 1,3 a 4,6 mm (média de 2,3 mm) e comprimento entre 28 e 84 mm (média de 50,6 mm). Em 87,8% dos casos, o pedículo irrigava o músculo grácil e apresentava vasos para a pele suprajacente.

**Conclusão:**

Com base nos dados encontrados, podemos concluir que é possível obter um retalho confiável da face medial do terço médio da coxa.

## Abstract (en)

**Objective:**

To analyze the anatomy of the perforating vessels irrigating the skin of the medial aspect of the thigh and originating from the secondary vascular pedicle of the gracilis muscle.

**Methods:**

We dissected 33 thighs from cadavers of both sexes to record and analyze the characteristics of the blood vessels irrigating the gracilis muscle in the middle third of the thigh.

**Results:**

All thighs (100%) had a secondary vascular pedicle in the intermuscular septum between the gracilis and vastus medialis/sartorius muscles, with branches irrigating the overlying skin on the medial aspect. The pedicle presented an arterial diameter ranging from 1.3 to 4.6 mm (mean: 2.3 mm) and a length ranging from 28 to 84 mm (mean: 50.6 mm). In 87.8% of the cases, the pedicle irrigated the gracilis muscle and presented vessels to the overlying skin.

**Conclusion:**

Based on the data found, we conclude that it is feasible to obtain a reliable flap from the medial aspect of the middle third of the thigh.

## Introdução

As lesões decorrentes de traumatismos de alta energia, oriundos principalmente de acidentes de trânsito, apresentam-se frequentemente com perda de partes moles que expõe estruturas nobres, como osso, tendões, vasos e nervos. 1 Elas representam situações de difícil manejo terapêutico, sendo um desafio para a cirurgião reconstrutivo.

O aperfeiçoamento das técnicas cirúrgicas tem aumentado os índices de sucesso no tratamento. Atualmente, os retalhos livres apresentam-se como uma opção eficaz entre as técnicas reconstrutivas. Os retalhos perfurantes são considerados uma evolução dos retalhos microcirúrgicos, por permitirem a obtenção de retalhos mais finos e, portanto, com melhores resultados funcional e estético. 2

Em 1967, Fujino 3 documentou a distribuição das artérias perfurantes para a nutrição do interstício, à semelhança das artérias axiais. Em 1988, Koshima et al. 4 foram pioneiros na utilização de retalhos baseados em artérias perfurantes formados exclusivamente por pele e tecido celular subcutâneo. Wei et al. 5 definiram que os retalhos baseados nas artérias perfurantes são aqueles nutridos por artérias que atravessam a fáscia profunda adjacente e que podem ser dissecadas através do músculo ou do septo intermuscular até o seu vaso de origem, sem a necessidade da inclusão do músculo no retalho.

Com interesse na face medial da coxa, alguns autores como Peek et al. 6 e Eom et al. 7 descreveram, em estudos anatômicos e relatos de casos, um retalho desta região, baseado nas artérias perfurantes provenientes do pedículo vascular principal do músculo grácil. Esse retalho foi amplamente estudado e pode ser utilizado na sua forma pediculada ou livre para a cobertura de diferentes defeitos na região inguinal, ou em regiões mais distantes.

Feng et al., 8 Scaglioni et al. 1 e Zheng et al. 9 descreveram, também por meio de estudos anatômicos e alguns relatos de casos clínicos, o retalho anteromedial da coxa ou medial inferior da coxa baseado em ramos perfurantes provenientes de artérias musculares que irrigam o músculo vasto medial. Em 1984, Song et al. 10 descreveram o retalho de perfurante da artéria femoral profunda, que pode também ser retirado na face posteromedial da coxa.

O músculo grácil, presente na região medial da coxa, é utilizado com frequência como retalho muscular livre funcional, e apresenta um padrão de irrigação classificado por Mathes e Nahai 11 como de tipo 2, no qual o músculo é irrigado por um pedículo vascular dominante, capaz de irrigar o músculo todo, mas também tem vários pedículos secundários menores que contribuem para sua irrigação. Alguns ramos desses vasos atravessam a fáscia profunda na região medial da coxa e irrigam o tecido subcutâneo e a pele suprajacente. Peek et al. 6 identificaram, no seu estudo anatômico com arteriografia, a presença de um pedículo secundário na região intermédia da coxa que tem múltiplas anastomoses intramusculares com o pedículo vascular principal.

O objetivo deste estudo foi avaliar os parâmetros anatômicos dos vasos que irrigam a pele e a fáscia da região medial da coxa, provenientes do pedículo vascular secundário do músculo grácil, no terço médio da coxa, definindo a sua localização, o diâmetro, o padrão de distribuição e as variações anatômicas, para mostrar que é possível obter um retalho confiável baseado neste pedículo vascular.

## Materiais e Métodos

Este estudo foi aprovado pelo Comitê de Ética da nossa instituição sob o número CAAE: 15621319.1.0000.5479.

Foi realizada a dissecção microcirúrgica de 33 coxas de 18 cadáveres, sendo 19 coxas provenientes de cadáveres de sexo masculino e 14, do sexo feminino. Foram registrados também dados biométricos do cadáver, como idade, altura e peso. Foram excluídos os cadáveres com doença vascular periférica conhecida como causa de morte, e aqueles com presença de cicatrizes na região da dissecção.

Todas as dissecções foram realizadas pelo mesmo cirurgião, com o auxílio de lupa cirúrgica com aumento de 3,5 vezes. Foram registrados parâmetros anatômicos do pedículo vascular, como comprimento e diâmetro dos vasos, localização, origem e distribuição, assim como sua relação com o comprimento da coxa. As medições foram feitas com fita milimetrada e paquímetro digital quadridimensional de 150 mm (Digimess Instrumentos de Precisão Ltda.).

Utilizou-se o teste de Kolmogorov-Smirnov para avaliar se as variáveis quantitativas do desfecho seguiam uma distribuição normal. As variáveis quantitativas foram analisadas pelos testes de igualdade de duas proporções e o de correlação de Pearson, e adotou-se o intervalo de confiança estatística de 95%.

## Dissecção

**Fig. 1:**
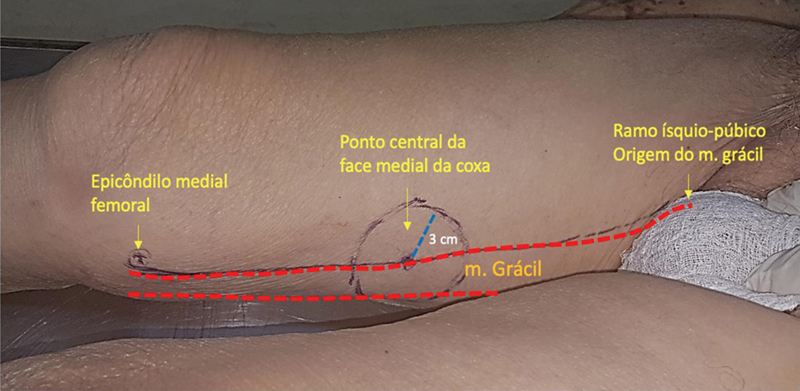
Face medial da coxa direita com desenho da localização do pedículo vascular.

**Fig. 2:**
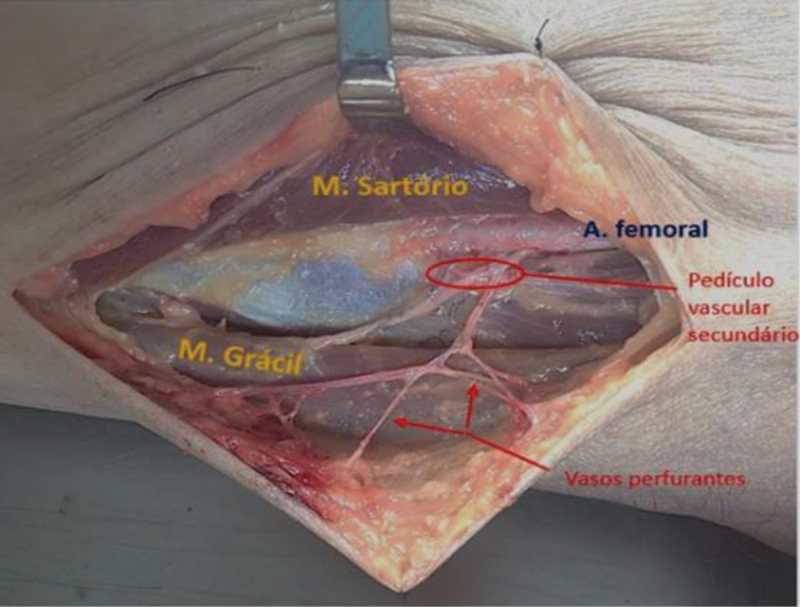
Dissecção do pedículo vascular secundário do músculo grácil com seus vasos perfurantes.

Inicialmente, traçou-se uma linha na face medial da coxa, desde o ramo isquiopúbico até o epicôndilo medial femoral ( Fig. 1 ). Essa linha coincide com a borda anterior do músculo grácil e, no ponto médio desta linha, foi desenhado um círculo com raio de 3 cm, dividido em quadrantes. Posteriormente, realizamos a incisão seguindo o contorno do círculo, e as artérias perfurantes foram identificadas por meio de divulsão delicada no tecido subcutâneo, e foram seguidas elas até o septo entre o músculo grácil e o músculo vasto medial/sartório. A maioria dos vasos identificados no septo intermuscular apresentava ramos musculares para o músculo grácil e, em poucos casos, para o músculo sartório. Esses vasos septais emitiam ramos perfurantes para irrigar a pele e o tecido subcutâneo suprajacente. A dissecção seguiu em profundidade até a artéria femoral, que foi identificada como a origem arterial em todos os casos ( Fig. 2 ). As veias, geralmente duas, eram tributárias da veia femoral, não da veia safena magna, que se encontrava próxima ao local de dissecção. O ramo que se dirige até o músculo, grácil ou sartório, foi ligado preservando-se a sua parte proximal, assim como as perfurantes que se dirigem até a pele, aumentando assim o comprimento dos vasos. Foram selecionados e documentados os vasos com diâmetro externo maior do que 0,5 mm.

## Resultados

A Tabela 1 mostra os dados antropométricos dos cadáveres dissecados, e na Tabela 2 são mostradas as características dos vasos do retalho.

**Tabela 1:** Dados antropométricos

Número da coxa	Idade (anos)	Lado	Sexo	Altura (cm)	Peso (kg)	Cumprimento da coxa (inguinal-côndilo medial femoral; cm)
**1**	56	Direito	Masculino	176	70	38
**2**	56	Esquerdo	Masculino	176	70	38
**3**	62	Direito	Masculino	174	66	36
**4**	62	Esquerdo	Masculino	174	66	36
**5**	56	Direito	Masculino	165	88	36
**6**	52	Direito	Masculino	172	78	35
**7**	52	Esquerdo	Masculino	172	78	35
**8**	56	Direito	Masculino	176	84	35
**9**	52	Direito	Masculino	150	43	35
**10**	52	Esquerdo	Masculino	150	43	35
**11**	64	Direito	Masculino	167	61	30
**12**	64	Esquerdo	Masculino	167	61	30
**13**	58	Direito	Masculino	170	70	30
**14**	58	Esquerdo	Masculino	170	70	30
**15**	59	Direito	Feminino	169	95	30
**16**	59	Esquerdo	Feminino	169	95	30
**17**	42	Direito	Masculino	182	76	35
**18**	42	Esquerdo	Masculino	182	76	35
**19**	66	Esquerdo	Masculino	175	74	26
**20**	58	Direito	Masculino	175	86	34
**21**	58	Esquerdo	Masculino	175	86	34
**22**	92	Direito	Feminino	164	68	28
**23**	92	Esquerdo	Feminino	164	68	28
**24**	47	Direito	Feminino	160	65	29
**25**	47	Esquerdo	Feminino	160	65	29
**26**	36	Direito	Feminino	165	70	33
**27**	36	E	Feminino	165	70	33
**28**	92	Direito	Feminino	163	55	32
**29**	92	Esquerdo	Feminino	163	55	32
**30**	85	Direito	Feminino	157	42	29
**31**	85	Esquerdo	Feminino	157	42	29
**32**	38	Direito	Feminino	160	62	29
**33**	38	Esquerdo	Feminino	160	62	29
**Média**	**57,8**			**162,5**	**66,5**	**31,3**
**Máximo**	**92**			**182**	**95**	**38**
**Mínimo**	**36**			**150**	**42**	**26**

**Tabela 2:** Características do pedículo vascular

Número da coxa	Cumprimento do pedículo (mm)	Diâmetro da artéria (mm)	Diâmetro das veias (mm)	Origem	Músculo irrigado	Quadrante	Variação
**1**	53	3,8	3	Femoral	Grácil	Anterossuperior	
**2**	48	3,4	3,2	Femoral	Grácil	Anterossuperior	
**3**	83	4,2	3,2	Femoral	Grácil	Anterossuperior	
**4**	76	4,6	3,6	Femoral	Grácil	Anterossuperior	
**5**	32	2,1	1,8	Femoral	Nenhum	Anteroinferior/Anterossuperior	Sem ramo muscular
**6**	41	3,6	3,4	Femoral	Grácil	Anteroinferior	
**7**	38	3,4	3,2	Femoral	Grácil	Anteroinferior	
**8**	63	3,2	2,8	Femoral	Sartório	Anterossuperior	Ramo para o sartório
**9**	38	2	2	Femoral	Grácil	Anterossuperior	
**10**	32	2,2	1,8	Femoral	Grácil	Anterossuperior	
**11**	32	1,3	1	Femoral	Grácil	Anterossuperior	
**12**	28	1,5	1,2	Femoral	Grácil	Anteroinferior	
**13**	78	1,6	1,4	Femoral	Grácil	Anterossuperior	
**14**	45	2,5	2	Femoral	Grácil	Anterossuperior	
**15**	84	1,8	1,8	Femoral	Grácil	Anterossuperior	
**16**	76	1,6	1,6	Femoral	Grácil	Anterossuperior	
**17**	38	1,3	1,2	Femoral	Grácil	Anterossuperior	
**18**	40	1,4	1,2	Femoral	Grácil	Anterossuperior	
**19**	32	1,6	1,4	Femoral	Grácil	Anterossuperior	
**20**	40	2,2	2	Femoral	Sartório	Anterossuperior	Ramo para o sartório
**21**	38	2	2	Femoral	Sartório	Anterossuperior	Ramo para o sartório
**22**	62	1,5	1,5	Femoral	Grácil	Anterossuperior	
**23**	60	1,3	1,5	Femoral	Grácil	Anterossuperior	
**24**	58	2	1,8	Femoral	Grácil	Anteroinferior	
**25**	61	2,4	1,9	Femoral	Grácil	Anteroinferior	
**26**	64	2,7	2,2	Femoral	Grácil	Anterossuperior	
**27**	68	2,4	2,3	Femoral	Grácil	Anterossuperior	
**28**	56	1,8	1,6	Femoral	Grácil	Anterossuperior	
**29**	42	2,8	2	Femoral	Grácil	Anterossuperior	
**30**	56	2,8	2,3	Femoral	Grácil	Anterossuperior	
**31**	58	2,5	2,2	Femoral	Grácil	Anterossuperior	
**32**	52	2	1,8	Femoral	Grácil	Anterossuperior	
**33**	50	1,8	1,6	Femoral	Grácil	Anterossuperior	
**Média**	**50,6**	**2,3**	**2,0**				
**Máximo**	**84**	**4,6**	**3,6**				
**Mínimo**	**28**	**1,3**	**1**				

**Fig. 3:**
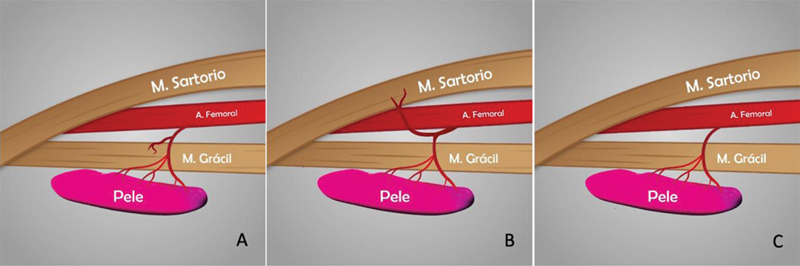
Padrões de distribuição arterial encontrados. Vasos com ramos para o músculo grácil ( **A** ). Vasos com ramos para o músculo sartório ( **B** ). Vasos perfurantes diretos, sem ramificação muscular ( **C** ).

Em todas as dissecções (100%), foi identificada pelo menos 1 artéria acompanhada de 2 veias, consideradas viáveis para realizar o retalho. Todos esses vasos foram localizados no septo intermuscular entre os músculos grácil e o vasto medial/sartório. Em 29 (87,8%) das 33 coxas, o vaso principal foi identificado como uma artéria septocutânea com ramificações para o músculo grácil; em 3 (9,1%) coxas, as artérias septocutâneas emitiam ramos para o músculo sartório; e 1 coxa (3%) apresentava 2 artérias septocutâneas diretas que não tinham ramos musculares ( Fig. 3 ).

**Fig. 4:**
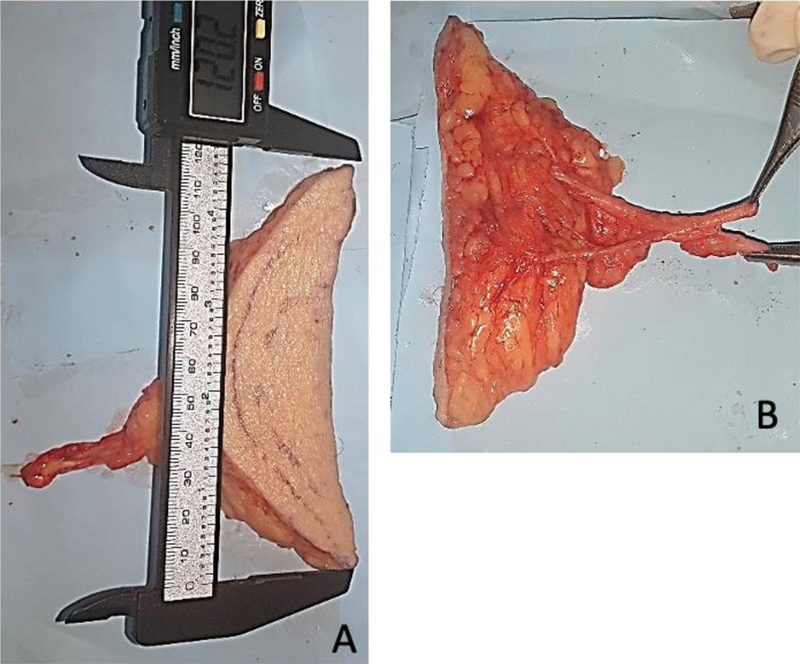
Retalho isolado com comprimento aproximado de 12 cm ( **A** ). Pedículo vascular ( **B** ).

Foi evidenciada, em todos os casos, pelo menos 1 artéria e 1 veia com diâmetro externo maior do que 0.5 mm, sendo o maior diâmetro encontrado de 4,6 mm, e o menor, de 1,3 mm. A média de todas as artérias foi de 2,3 mm. Em geral, foram identificadas duas veias por cada artéria. O maior diâmetro entre as veias foi de 3,6 mm, e o menor, de 1.0 mm, com uma média de 2,0 mm ( Fig. 4 ). O comprimento dos vasos, desde a sua origem nos vasos femorais até as ramificações menores no subcutâneo, variou entre 84 mm e 28 mm, com uma média de 53,2 mm.

A análise estatística foi iniciada com a aplicação do teste paramétrico de Kolmogorov-Smirnov para avaliar a normalidade da distribuição das variáveis quantitativas, e concluímos que a distribuição era normal. Na distribuição da frequência relativa (percentuais) dos fatores qualitativos, aplicando o teste da igualdade de duas proporções, observamos que o pedículo estava localizado no quadrante anterossuperior em 84,8% das coxas dissecadas, bem como o grácil era o músculo mais comumente irrigado por este pedículo em 87,9% dos casos ( *p* < 0,001), ambos dados mostrados nas Tabelas 3 4 respectivamente.

**Tabela 3:** Localização do pedículo no quadrante anteroinferior e anterossuperior

		n	%	Valor de *p*
**Quadrante**	Anteroinferior Anterossuperior	5 28	15,2% 84,8%	< 0,001

**Tabela 4:** Músculos irrigados pelo pedículo vascular

		n	%	Valor de *p*
**Músculo irrigado**	Grácil Nenhum Sartório	29 1 3	87,9% 3,0% 9,1%	< 0,001 < 0,001

**Fig. 5:**
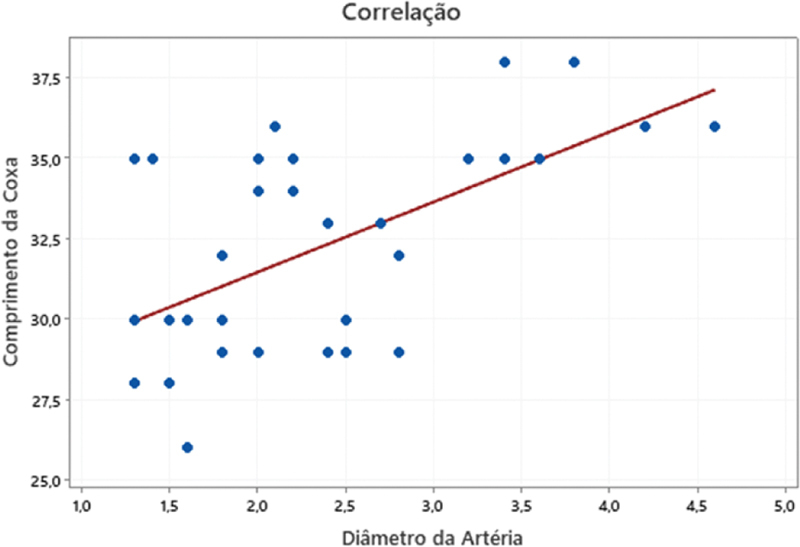
Relação entre comprimento da coxa e diâmetro da artéria.

Na análise da correlação entre essas variáveis, utilizando o teste de Pearson, observou-se significativa associação diretamente proporcional entre o diâmetro dos vasos e o comprimento da coxa ( *p* < 0,001), como mostrado na Fig. 5 .

## Discussão

Como descrito por Giordano et al. 12 e Peek et al., 6 o músculo grácil recebe irrigação a partir de um pedículo principal, proximal, originado da artéria circunflexa femoral medial e pelo menos dois pedículos secundários distais provenientes da artéria femoral ou da artéria poplítea. A maioria desses pedículos, primários e secundários, apresentam ramos que perfuram a fáscia profunda e irrigam um território considerável de pele sobre o músculo grácil, correspondente a pelo menos o dobro da largura do músculo grácil. As características anatômicas relacionadas ao pedículo principal já foram bem estudadas, e temos parâmetros confiáveis para a realização de retalhos musculares ou musculocutâneos do grácil. No entanto, os pedículos secundários que poderiam ser usados para retalhos fasciocutâneos ainda não estão bem estabelecidos.

Os retalhos fasciocutâneos proximais da coxa, que são irrigados por ramos do pedículo principal do músculo grácil, são amplamente utilizados na sua forma pediculada e livre. Entretanto, comprometem o pedículo principal do músculo grácil e requerem cuidado com os ramos do nervo obturador que estão presentes no local da disseção. O retalho do pedículo vascular principal, que é retirado da região medial proximal da coxa, representa uma área mais susceptível à contaminação e de acesso limitado para realizar os curativos no pós-operatório. 6

Scaglioni et al. 1 descreveram alguns retalhos da região anteromedial na região distal da coxa, com base nos vasos musculocutâneos perfurantes que atravessam o vasto medial. No entanto, é necessária dissecação intramuscular para se obter um pedículo de comprimento adequado, e a área da cicatriz fica mais exposta quando comparada com a do retalho do pedículo principal. Song et al. 10 e Algan e Tan 13 descreveram um retalho confiável, proveniente de perfurantes da artéria femoral profunda, que também pode ser obtido nesta região; porém, muitas vezes, o posicionamento do paciente inviabiliza a sua obtenção.

O retalho proposto está localizado na face medial do terço médio da coxa e, após a sua retirada, o fechamento primário da área doadora pode ser realizado sem necessidade de enxerto cutâneo. A cicatriz fica em uma região de pouca exposição. A área doadora fica mais distante das regiões inguinal e perineal quando comparada à do retalho do pedículo principal, e é mais confortável para a troca de curativos e mais confiável para se evitar contaminação e infecções.

O diâmetro médio das artérias estudadas neste trabalho foi de 2,3 mm, valor que, segundo Jacobson e Suarez, 14 é seguro para anastomoses vasculares em retalhos livres. O comprimento médio do pedículo foi de 50,6 mm, e mostrou-se adequado para retalhos livres ou pediculados; porém, em uma das dissecções, foi identificado um pedículo menor do que 30 mm (28 mm), relativamente curto, que dificultaria a anastomose e dependeria da área e do vaso receptor.

Alguns parâmetros interessantes encontrados na dissecção foram: todos os pedículos vasculares foram localizados na região central da face medial da coxa, no interior de um desenho circular de 6 cm de diâmetro, cujo ponto central coincidia com a borda anterior do músculo grácil; a origem arterial foi localizada, em 87,8% dos casos, no quadrante anterossuperior do círculo, e a maioria dos vasos encontrados (87,8%) correspondiam a vasos septocutâneos, com ramos para o músculo grácil. Deve-se ter cuidado com a veia safena maior, que, muitas vezes, fica incluída no desenho do retalho, e deve ser ligada e incluída no retalho diante da impossibilidade de isolá-la.

**Fig. 6:**
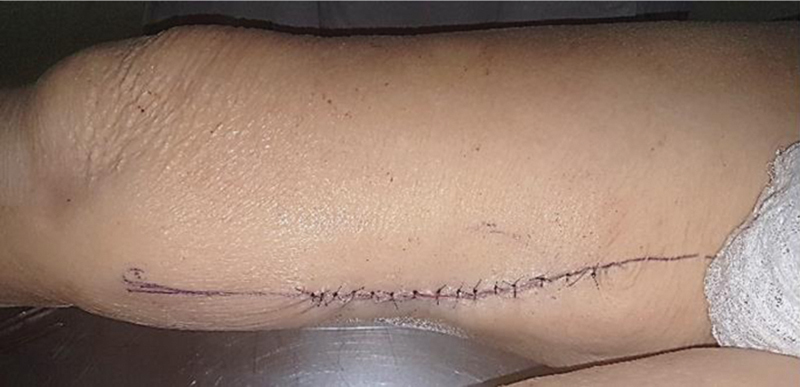
Fechamento primário da área doadora.

Segundo Giordano et al., 12 a largura da pele irrigada pelas perfurantes corresponderia, no mínimo, ao dobro da largura do músculo grácil subjacente. A área doadora poderia ser fechada primariamente, com relativa facilidade, sem necessidade de enxertia de pele ( Fig. 6 ).

Os parâmetros encontrados permitirão realizar a dissecção e a retirada de um retalho microcirúrgico com segurança. A pele da região medial da coxa é mais fina e versátil. O retalho dessa região é apropriado para a cobertura de defeitos de pequeno e médio portes (com diâmetro menor do que 15 cm).

Estudos de séries de casos realizados nos últimos anos 15 16 mostram as vantagens de se usar retalhos de vasos perfurantes para a obtenção de retalhos menos volumosos e maleáveis; porém, o retalho aqui apresentado não foi descrito em nenhuma desses trabalhos.

**Fig. 7:**
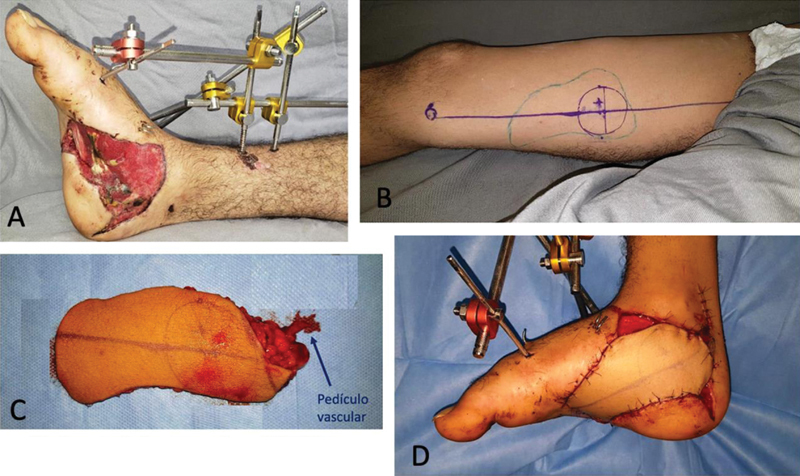
Caso operado. Pé direito com defeito de cobertura na face inferomedial ( **A** ). Face medial da coxa direita com desenho do retalho e localização do pedículo vascular ( **B** ). Retalho isolado ( **C** ). Cobertura do defeito com anastomose nos vasos tibiais posteriores ( **D** ).

**Fig. 8:**
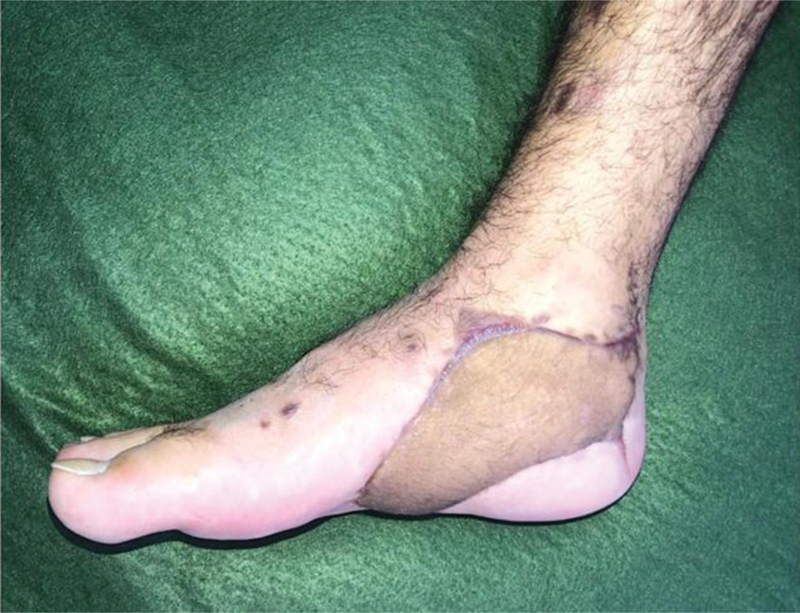
Caso operado aos três meses de acompanhamento no pós-operatório.

Para demonstrar a viabilidade do retalho, realizamos uma cirurgia em um paciente jovem, do sexo masculino, vítima de acidente de trânsito que, além de múltiplas fraturas no pé direito, apresentava perda da pele na sua face medial, que se estendia até a face plantar e expunha os tendões e a aponeurose plantar ( Fig. 7 ). Foi realizada cobertura microcirúrgica com o retalho descrito, com anastomose dos seus vasos em ramos e tributárias dos vasos tibiais posteriores. O procedimento foi bem-sucedido, de modo que se evidenciou a viabilidade da técnica ( Fig. 7 e Fig. 8 ).

Acreditamos que uma das melhores indicações para este retalho seria a cobertura cutânea do membro superior, que requer pele fina e maleável, e os vasos doadores são de menor calibre. A nossa intenção é realizar um estudo de série de casos utilizando esse tipo de retalho para publicação posterior dos resultados, mostrando os pontos positivos e negativos.

## Conclusão

O pedículo secundário do músculo grácil, que emite ramos septocutâneos e perfurantes para a pele da região medial da coxa, foi encontrado em todas as coxas dissecadas (100%), e mostrou-se confiável para a obtenção de um retalho fasciocutâneo desta região.
